# Design methodology of a promising category of metal phosphate electrodes for quasi-solid-state proton batteries

**DOI:** 10.1093/nsr/nwaf226

**Published:** 2025-05-31

**Authors:** Yijun Zhong, Leqi Zhao, Daqin Guan, Zehua Wang, Hongwei Wu, Jingyuan Liu, Zongping Shao

**Affiliations:** Curtin Centre for Advanced Energy Materials and Technologies, WA School of Mines: Minerals, Energy and Chemical Engineering (WASM-MECE), Curtin University, Perth, WA 6102, Australia; Curtin Centre for Advanced Energy Materials and Technologies, WA School of Mines: Minerals, Energy and Chemical Engineering (WASM-MECE), Curtin University, Perth, WA 6102, Australia; Curtin Centre for Advanced Energy Materials and Technologies, WA School of Mines: Minerals, Energy and Chemical Engineering (WASM-MECE), Curtin University, Perth, WA 6102, Australia; Curtin Centre for Advanced Energy Materials and Technologies, WA School of Mines: Minerals, Energy and Chemical Engineering (WASM-MECE), Curtin University, Perth, WA 6102, Australia; Curtin Centre for Advanced Energy Materials and Technologies, WA School of Mines: Minerals, Energy and Chemical Engineering (WASM-MECE), Curtin University, Perth, WA 6102, Australia; Altech Batteries Ltd, Subiaco, WA 6008, Australia; Curtin Centre for Advanced Energy Materials and Technologies, WA School of Mines: Minerals, Energy and Chemical Engineering (WASM-MECE), Curtin University, Perth, WA 6102, Australia

**Keywords:** proton battery, transition metal phosphate, solid-state battery, redox couple, material design

## Abstract

A proton battery is a low-cost and safe alternative to a Li-ion battery. However, limited electrode options, especially cathodes, stably perform in acidic electrolytes and many of them show large voltage decreases along with the depth of discharge. Herein, an all-phosphate configuration is proposed to open up an avenue for a promising category of metal phosphate electrodes. Fe^2+/3+^ and Mn^2+/3+^ are investigated, for the first time, as example redox couples of phosphates for anode and cathode, respectively. The dynamic electronic and structural variations during the proton (de)-insertion process are captured using *in-situ* X-ray absorption spectroscopy (XAS). *In-situ* distribution of relaxation times (DRT) analysis reveals that the insufficient charge transfer hinders the performance, which is optimized by forming a phosphate-carbon composite. An NH_4_MnPO_4_·H_2_O-carbon composite presents a capacity of 121 mAh g^−1^ with flat voltage profiles and excellent high-rate performance at 150 A g^−1^. A full quasi-solid-state proton battery demonstrates smooth operation for 5000 cycles.

## Teaser

This work addresses the missing methodologies for developing a category of transition metal phosphate materials for rechargeable proton (H^+^) batteries.

## INTRODUCTION

Rechargeable batteries are important for realising the transition from conventional fossil fuel energy to green and renewable energy sources (e.g. wind, solar, tidal). For electric applications that require both high energy density and high-power output, the power supply system usually requires two or more individual energy storage units, i.e. a main unit (e.g. Li-ion battery) and an auxiliary unit that provides an instant high-power output. The Li-ion battery is the most successful commercially available battery which usually provides a sufficient energy density as the main unit for energy storage; however, it has limited capability of instant power output [1–4]. Contrarily, electrical double-layer capacitance provides instant high-power outputs; however, it has a very low energy density [5,6]. From an engineering point of view, a system with fewer units could reduce the chance of malfunctioning. Therefore, an energy storage system with a single unit that has a balance between energy density and power output is highly demanded.

Proton (H^+^) has the smallest ionic radius among cations and therefore is a promising charge carrier alternative to conventional cations like Li^+^, Na^+^ and Zn^2+^ [7–9]. In a wide definition, a battery with a proton as a major charge carrier to trigger electrochemical reactions on both electrodes is called a proton battery [10]. Proton batteries have a significant advantage over Li-ion batteries regarding their capability for ultra-fast charging and discharging. Also, proton batteries with an aqueous acidic electrolyte can operate at ultra-low temperatures, which is almost impossible to achieve for Li-ion batteries with commercial organic electrolytes. Proton batteries show promise for providing a good balance between energy density and power output, however research on proton batteries is still in the early stages. In previous research, intensive efforts were made to develop and optimize electrolytes and electrodes. A variety of options for anodes have been developed with high capacity and good cycling stability [11,12] but limited options for cathodes with high capacity and low voltage decrease along with the depth of discharge. Polymers [13] and metal-organic frameworks [14] (e.g. Turnbull's blue analogue (TBA) or Prussian blue analogue (PBA)) are representative types of cathodes for proton batteries. Most of them either had fair theoretical capacity (<100 mAh g^−1^) or had relatively low voltages (<0.8 V vs standard hydrogen electrode, SHE, with significant voltage drop dependent on the depth of discharge) [14,15]. Though some other examples are reported as having high-capacity and relatively high voltage ∼1 V vs SHE (e.g. LiVPO_4_F [16,17] and 2,5-dichloro-1,4-phenylene bis((ethylsulfonyl)amide) (HDC) [18]), options are still very limited for cathodes with high output voltage, high capacity, good stability and good high-rate performance. The development of a series of new cathodes is urgently required. The challenge is partially because of the limitation of the narrow potential window of water splitting which is a competitive electrochemical process that should be avoided. The challenge of developing new materials is also due to the poor stability of most metal compounds in acidic electrolytes [19]. This could be associated with the high solubility and fast diffusion of active transition metal cations (e.g. Mn^2+^) from the electrode to the electrolyte. Unfortunately, most metal oxides (e.g. MoO_3_, TiO_2_) [20–22] that are relatively stable in acidic electrolytes usually fall in lower potential ranges (∼0 V vs SHE) that are usually suitable as anodes for proton batteries.

Olivine-type metal phosphate compounds, e.g. LiFePO_4_ or LiFe_1−x_Mn_x_PO_4_ have been investigated as cathodes for Li-ion batteries [5,23,24]. These compounds present capacities close to their theoretical values (∼170 mAh g^−1^) in non-aqueous and they also demonstrate good performance in aqueous Li-ion batteries. During charging and discharging, the variation of the valence state of the transition metal, e.g. Fe^2+/3+^ provides the capability for energy storage, accompanied by the Li^+^ (de-)insertion process. It is possible to utilize these Li-containing olivine-type metal phosphate compounds for proton batteries. It is worth mentioning that one of the main purposes of developing proton battery technology is to avoid the utility of expensive and limited Li resources. Therefore, transition metal phosphates with other cations (e.g. H^+^ and NH_4_^+^) are more promising candidates as an initial cathode material for proton batteries. Despite the transition metal phosphates for proton batteries and lithium transition metal phosphates for Li-ion batteries sharing some similarities regarding theoretical capacities and composition, the detailed battery chemistries of these candidates have not been investigated in detail previously in proton batteries.

It is worth mentioning that metal phosphates are a large category of materials with different transition metal cations that have promising capabilities for electrochemical proton storage. Utilizing phosphate electrodes in phosphoric acid electrolytes could also alleviate the dissolution of transition metal electrodes. Based on the concept of the solubility product constant (*K_sp_*), the solubility of the metal compounds (e.g. MPO_4_) is associated with the concentration of both cations (e.g. M^3+^) and anions (e.g. PO_4_^3−^). In a previous study on proton batteries and research in rechargeable Zn–MnO_2_ batteries that have acidic electrolytes, adding additional active transition metal cations (e.g. Mn^2+^) to the electrolyte can alleviate the dissolution of the MnO_2_ electrode [25]. However, because of the low utilization rate of the additional cations in the electrolyte, this strategy may reduce the energy density of the battery. Besides cations, the concentration of anions may also influence the dissolution of the electrode. For oxide and hydroxide materials, an increase in the concentration of anions (O^2−^/OH^−^) in an electrolyte results in a higher pH value and lower concentration of protons (H^+^) in the electrolyte, which decreases the proton conductivity in the electrolyte and may also negatively affect proton diffusion through the porous electrode layer. For phosphate materials, utilizing an all-phosphate configuration of a battery (i.e. phosphate electrodes and a high-concentration phosphoric acid electrolyte) could take advantage of the high concentrations of protons and phosphate ions. The former ensures efficient proton diffusion and increases the kinetics of the proton-involved electrochemical reactions. The latter, i.e. phosphate ions, alleviate the dissolution of the metal phosphates. It is important to mention that many transition metal phosphates show low solubility in aqueous solutions and have low *K_sp_* values (e.g. FePO_4_·2H_2_O: *K_sp_* = 9.91 × 10^−16^, Co_3_(PO_4_)_2_: *K_sp_* = 2.05 × 10^−35^) [26]. This provides a good opportunity for exploring a wider range of phosphate electrodes. Despite some examples of metal phosphates being reported (e.g. LiVPO_4_F as cathode [16,17], Mo phosphate as anode [27]) for proton batteries, more electrode options are yet to be discovered and investigated. A methodological guidance for phosphate electrode material development and cathode-anode matching is missing.

The objective of this work is to provide methodologies for the fast screening, design, synthesis and evaluation of promising transition metal phosphates as both cathode and anode and for matching them in a full proton battery. The following aspects are considered when screening for suitable cations for phosphate electrodes in proton batteries. The primary criterion is that the redox potential of the cation should, overall, fall into the stable window of water to avoid the competitive hydrogen evolution reaction (HER) and oxygen evolution reaction (OER). Subject to the primary criterion, a consideration is that the potential gap between the two redox couples of cathode and anode should be as large as possible because it provides a higher voltage and thereby a higher energy density for the full proton battery. The secondary criterion is the stability of the redox states during proton insertion and de-insertion. For example, the Cu^+/2+^ redox couple has electrochemical potentials above the HER and is suitable as anodes. However, the Cu element is not stable in +1 valence as a phosphate. During reduction, Cu^2+^ is more likely to be further reduced to 0 valence (metallic Cu). The redox between Cu and Cu^2+^ is a typical reaction of a metal electrode rather than proton insertion and de-insertion. Other aspects should also be considered in addition to the above two criteria. First, cations of lighter elements are preferred because they offer higher theoretical capacities for the same amount of proton storage. For example, transition elements in Period 4 (e.g. V, Cr, Mn, Fe, Co, Ni) are more favourable than those from Period 5 (e.g. Mo, Ru, Ag) and 6 (e.g. W, Ir). Second, the cation elements should ideally be low-cost and abundant.

In this work, based on the above criteria and considerations, Mn^2+/3+^ and Fe^2+/3+^ were selected as representative redox couples as cathode and anode materials, respectively, for rechargeable proton batteries. Upon optimization of the charge transfer by forming a phosphate-carbon composite, a composite cathode material containing NH_4_MnPO_4_·H_2_O presents high capacity with a flat voltage profile and excellent high-rate performance. To address the instability of phosphate materials in acidic environments, a proton battery design with an all-phosphate configuration utilizing the developed phosphate electrodes and a quasi-solid-state electrolyte is proposed for stable operation of the battery.

## RESULTS AND DISCUSSION

### Screening, design, synthesis and preliminary validation of example phosphate electrodes

**Figure 1.**
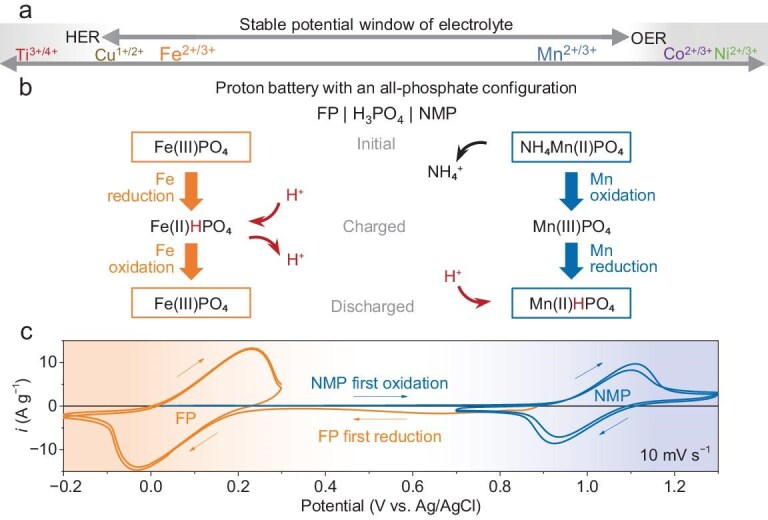
(a) Redox couples of phosphates with different cations and their relevant redox potential levels. Onset potential levels of HER and OER which are unfavourable side reactions, are also included as a reference. (b) Illustrative diagram of the design of proton battery with an all-phosphate configuration with FP and NMP as example anode and cathode and phosphoric acid as electrolyte. (c) CV scans of FP electrode and NMP electrode with a 3-electrode setup.

As described in the Introduction, the primary criterion for evaluating the suitability of the phosphates in a proton battery is their redox potential [19]. Most state-of-the-art proton batteries have an aqueous electrolyte with a concentrated acidic environment. This draws an overall potential range with lower (∼0 V vs SHE) and upper (∼1.2 V vs SHE) limits within the stable potential window of water (0 to 1.2 V vs SHE). Prediction of the potential (vs Li^+^/Li) of the many transition metal phosphates (MPO_4_, example M = Fe, Mn, Co, Ni, Cu) with different cation redox couples (e.g. Fe^2+/3+^, Fe^3+/4+^, Mn^2+/3+^) have been previously reported [28,29]. The redox potentials of some example phosphates have been investigated in Li-ion batteries, confirmed with experimental data (e.g. Fe, Mn, Co, Ni phosphates) [30]. Though these potentials are referred to as Li^+^/Li, they do provide good guidance on the approximate value versus SHE, which is valuable for a fast screening of promising candidates. For example, based on the M^2+/3+^ redox, the LiFePO_3_, LiMnPO_3_, LiCoPO_3_ and LiNiPO_3_ cathodes present redox potentials at 3.5, 4.1, 4.8 and 5.2 V vs Li^+^/Li, respectively, in Li-ion batteries [28,29]. They could present approximately potential levels of 0.3, 0.9, 1.6 and 2.0 V vs SHE. The screening first excluded those phosphate materials that may have redox couples (e.g. Ti^3+/4+^, V^2+/3+^) [28] with low potentials which may trigger the HER during reduction (discharging) and have redox couples (e.g. Mn^3+/4+^, Fe^3+/4+^) [28] with high potentials which may trigger the OER during oxidation (charging). It is worth mentioning that these redox couples may still have the possibility of being utilized in the future upon the successful development of an electrolyte with a wider stable voltage window. As highlighted in Fig. 1a, redox couples that have a redox potential below but near the onset of OER could be a good candidate for the cathode, for example, Mn^2+/3+^. Similarly, those redox couples (e.g. Fe^2+/3+^) that have potential above the HER, and at the same time will form a high enough potential gap to the cathode, are favourable. This is important for the matching of the phosphate cathode and phosphate anode, which provides a sufficiently high operating voltage output as a full proton battery. In this work, Mn^2+/3+^ and Fe^2+/3+^ were chosen as the example redox couple, respectively, for the cathode and anode.

To further match the cathode and anode, suitable initial forms of metal phosphates and charge states were then designed. The cathode material with the Mn^2+/3+^ redox couples was synthesized in the form of its uncharged state, i.e. Mn^2+^ (Fig. 1b). The initial cathode material will ideally be MnHPO_4_ or its hydrated forms (i.e. MnHPO_4_·xH_2_O) where Mn has an initial valence state of 2+. Our attempts at synthesis using a participation method (by combining Mn^2+^ and PO_4_^3−^) found it difficult to obtain MnHPO_4_ or its hydrated form. Therefore, an alternative component that replaces the H^+^ with NH_4_^+^ is adopted (i.e. NH_4_MnPO_4_·H_2_O, denoted below as NMP). Based on the full redox between 2+ and 3+ of the Mn element and the molar mass of NMP (186 g mol^−1^), it has a theoretical capacity of 144 mAh g^−1^. The Fe^2+/3+^ redox couple for the anode was synthesized in the form of a charged state, i.e. Fe^3+^ (Fig. 1b). The initial anode material will ideally be FePO_4_ or its hydrated forms (i.e. FePO_4_·xH_2_O). In this paper, FePO_4_·2H_2_O (denoted below as FP) is easily synthesized via a participation method. Based on the full redox between 2+ and 3+ of the Fe element and molar mass of FePO_4_·2H_2_O (187 g mol^−1^), it has a theoretical capacity of 143 mAh g^−1^. The NMP and FP are both stable in ambient air and room temperature. The synthesis method is similar to the commercial preparation of Li-free precursors for LiFePO_4_ and LiFe_1−x_Mn_x_PO4 electrode materials. This could make them easily adaptable for large-scale production. Cyclic voltammetry (CV) scans in a 3-electrode cell with 85% H_3_PO_4_ (Fig. 1c) verified the potentials of the FP (anode material) and NMP (cathode material), which present a couple of oxidation-reduction peaks centred at 0.1 and 1.0 V vs Ag/AgCl, respectively. The matching of FP and NMP in a full proton battery is thereby expected to have a maximum voltage output of ∼0.9 V, which was achieved in a full battery as discussed in a later section.

### Characterizations of the example anode material and cathode material

**Figure 2.**
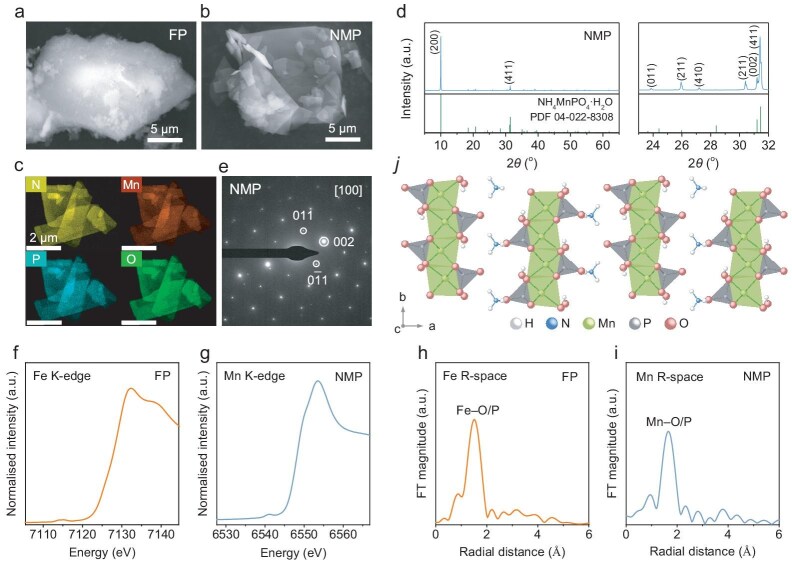
SEM images of (a) FP and (b) NMP. (c) EDS elemental mapping of NMP. (d) XRD profile of NMP. (e) SAED pattern of NMP. XANES spectra of (f) FP and (g) NMP, the *k*^3^-weighted EXAFS spectra of (h) FP and (i) NMP. (j) Illustrative demonstration of the crystal structure of NMP.

Further characterizations were performed to provide detailed information on the FP and NMP materials. The scanning electron microscope image (Fig. 2a) indicated that the FP nanoparticles are agglomerated into larger secondary particles. FP has a high specific surface area (103.7 m^2^ g^−1^) and a total pore volume (0.248 cm^3^ g^−1^) as revealed by N_2_ adsorption/desorption analysis (Fig. S1). The X-ray photoelectron spectroscopy (XPS) survey spectrum of FP (Fig. S2) indicates it contains Fe, P and O elements. Thermal gravimetric analysis (TGA) indicates the loss of water after being heated, which suggests that FP has a formula of FePO_4_·2H_2_O (Fig. S3). NMP presents a morphology of stacking thin sheets (Fig. 2b). It shows a non-porous feature (Fig. S4) with a low specific surface (1.7 m^2^ g^−1^) and a negligible pore volume (0.003 cm^3^ g^−1^). Elemental mapping of the NMP nanosheet indicates the homogeneous distribution of N, Mn, P and O elements (Fig. 2c), which aligns with the XPS survey spectrum of NMP (Fig. S5). TGA further confirms that NMP has a formula of NH_4_MnPO_4_·H_2_O with an NH_4_^+^ group and one molecular water (Fig. S6).

The crystal structures of the FP and NMP were analyzed with XRD. FP has an amorphous character (Fig. S7) while NMP presents a well-defined crystalline feature (Fig. 2d). The XRD profile of NMP (Fig. 2d) matches the simulated peak from the PDF card of orthorhombic NH_4_MnPO_4_·H_2_O (No. 04–022–8308). The selected area electron diffraction (SAED) pattern (Fig. 2e) of the NMP with a zone axis of [1 0 0] presents the (0 1 1), (0 0 2) and (0 − 1 1) planes with lattice distances of 3.70, 2.84 and 3.70 Å, respectively. The SAED result confirms the orthorhombic structure of the NMP materials and aligns with the XRD peak analysis.

The local electronic structures of FP and NMP were investigated using Fe and Mn K-edge X-ray absorption near-edge structure (XANES) spectra (Fig. 2f and g). The XANES spectra of FP (Fig. 2f) and NMP (Fig. 2g) show a strong resemblance to those reported in the literature [31,32], indicating the presence of an M–PO_4_ structure. Additionally, the local atomic structure was analyzed through Fe and Mn K-edge extended X-ray absorption fine structure (EXAFS) spectra. As shown in Fig. 2i, the Mn EXAFS spectrum primarily reveals Mn–O/P bonds, consistent with the MnO_6_ layered structure depicted in Fig. 2j. Similar findings are also observed in the Fe EXAFS spectrum of FP (Fig. 2h).

### Investigation of dynamic changes in the electronic structures of the anode and the cathode

**Figure 3.**
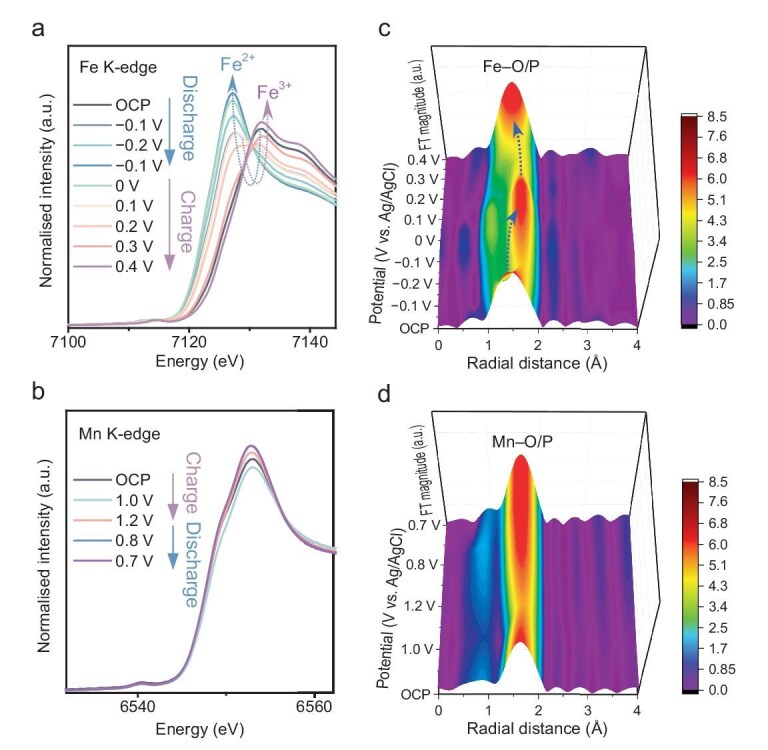
*In-situ* X-ray absorption (a) Fe K-edge of the FP electrode and (b) Mn K-edge of the NMP electrode. 3D *in-situ* EXAFS spectra of (c) Fe K-edge of the FP electrode and (d) Mn K-edge of the NMP electrode.

*In-situ* XANES spectra (Fig. 3) were obtained under concentrated phosphoric acid conditions (Fig. S8) to gain insights into the electronic structure and local geometry of the FP anode and the NMP cathode. As shown in Fig. 3a, the edge position of Fe in FP gradually shifts to lower energies during discharging, indicating a reduction in the Fe electronic structure [31]. Notably, during the charging process, the Fe edge position gradually shifts back to higher energies along with the potential increase, eventually returning to a spectrum almost identical to the open circuit potential (OCP) state. This suggests that Fe undergoes changes in its valence state during the charging and discharging processes, exhibiting good reversibility. In contrast, the Mn XANES in NMP exhibited smaller changes, with a slight increase in the white line intensity during charging (Fig. 3b). During discharging, the white line intensity slightly decreased, which is likely related to the migration of H⁺. These phenomena indicate that a limited portion of the active Mn in the anode surface exhibits valence state changes. To further confirm the key role of FP and NMP materials, *in-situ* EXAFS measurements were performed to reveal the local geometry of the FP anode and the NMP cathode, individually, during charging and discharging. As shown in Fig. 3c and d, the *in-situ* EXAFS spectra of the FP anode exhibit a different behaviour compared to the Mn cathode. The Fe EXAFS shows an increase in bond length during discharge, which returns to the original bond length during charging, consistent with the previously mentioned *in-situ* XANES results. In contrast, the Mn cathode shows insignificant changes, implying that only limited surface H⁺ interaction/adsorption may occur.

*Ex-situ* XPS was applied to provide supplementary information about the evolution of the chemical and electronic structure. As presented in Fig. S9, the Fe element in the initial FP has a valence state predominantly of +3 [33,34]. After a discharging (reducing) process, the Fe was proportionally converted to 2+. In a subsequent charging (oxidation) process to complete a full cycle, the proportion of Fe^2+^ decreased along with the increase in Fe^3+^. The evolution of the Fe valence states validates the electrochemistry based on the Fe^2+/3+^ redox couple. The comparison of Fe valence states between the initial FP and after 1 cycle (at the charged state) indicates that the surface Fe redox was not fully reversible. After the 1st cycle, the Fe valence state did not show a significant difference at the charged state in the subsequent 2nd cycle and 20th cycle. The Mn element in the initial NMP (Fig. S10) has mixed valence states between +2 and +3 [35,36], where +2 is predominant. The *ex-situ* XPS validates the electrochemistry based on Mn^2+/3+^ redox coupling. Similar to FP, the redox of surface Mn in NMP was not fully irreversible.

### Identifying the bottleneck of electrochemical redox

**Figure 4.**
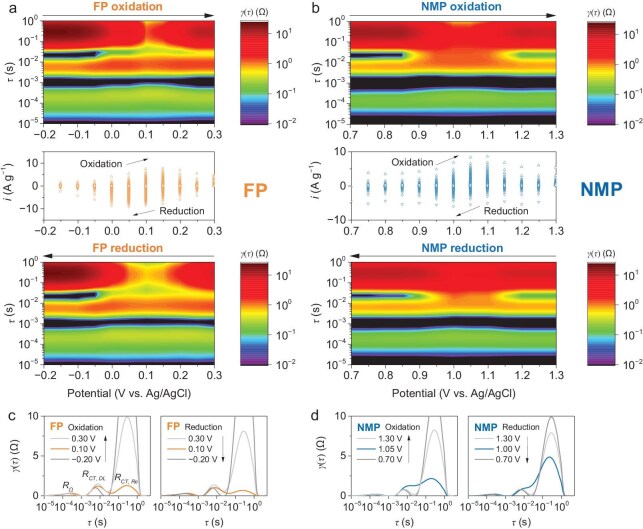
DRT analysis of SPEIS impedance with a 3-electrode configuration of (a) the FP electrode and (b) the NMP electrode. DRT profiles of redox-active potentials during charging and discharging of (c) the FP electrode and (d) the NMP electrode.

To investigate the resistance of different electrochemical processes of the phosphate electrodes at different potentials, *in-situ* staircase potentio electrochemical impedance spectroscopy (SPEIS) technique was applied with an interval potential of 50 mV in the voltage windows relevant to the redox of FP (−0.2 to 0.3 V, vs Ag/AgCl, Fig. 4a) and NMP (0.7 to 1.3 V, vs Ag/AgCl, Fig. 4b), respectively. As indicated in the current density–potential (*i*–*V*) profiles (Fig. 4a and b), the electrode was charged/discharged at a constant potential level for 1 min where equilibrium was established at this potential (as reflected by the current density drop close to 0). The Nyquist plots demonstrated heavy overlaps of resistance contributions from different electrochemical processes. Distribution of relaxation time (DRT) analysis was applied to reveal the major resistance contributions in the phosphate electrodes. DRT heat maps (Fig. 4a and b) were used to better demonstrate the variations of resistance during these processes. With different responses to time constant (*τ*) with the potential variation range (±10 mV), they can be attributed to ohmic resistance from the electrolyte and contact of different components (*R_Ω_, τ* range from 10^−5^ to 10^−3^ s), charge transfer resistance relevant to double-layer capacitance (*R_CT, DL_, τ* range from 10^−3^ to 10^−2^ s), and charge transfer resistance relevant to the redox reactions of active cations (*R_CT, Re_, τ* range from 10^−2^ to 10^0^ s).

The *R_Ω_* was maintained at a low level in the entire evaluated potential range for oxidation and reduction for both the FP electrode (0.3 Ω, Fig. 4a and Figs S11, S12) and the NMP electrode (0.2 Ω, Fig. 4b and Figs S13, S14). The *R_Ω_* has an insignificant change during the entire potential range because of the large volume of electrolyte (20 mL) and sufficient contact between the liquid electrolyte and the working electrode. Similarly, the *R_CT, DL_* presents low-level and relatively stable values in the entire potential range for both redox directions for the FP electrode (1.0–1.5 Ω) and the NMP electrode (0.8–1.3 Ω). The results of the *R_CT, DL_* reflect the characteristics of the double-layer capacitance process.

The major variation of the resistance is attributed to the *R_CT, Re_*, which is highly related to the redox of Fe^2+/3+^ and Mn^2+/3+^. Take the FP electrode as an example, in the reduction direction, a high value of *R_CT, Re_* (>14 Ω) is observed in the voltage range <0.05 V (vs Ag/AgCl) as the insufficient potential to trigger the oxidation of Fe^2+^ to Fe^3+^. The *R_CT, Re_* sharply decrease at a potential higher than 0.05 V and reaches a minimum value of 1.3 Ω at 0.10 V (Fig. 4a and c). As potential further increases after 0.10 V, the *R_CT, Re_* increases again due to the insufficient reactant (Fe^2+^). As suggested from the *i*–*V* profile (middle image of Fig. 4a) the highest response current is reached at 0.10 V, indicating the maximum oxidation reaction of Fe^2+^ to Fe^3+^. Therefore, the minimum *R_CT, Re_* during oxidation is an important parameter that reflects the kinetics of Fe^2+^ oxidation. Likewise, minimum *R_CT, Re_* (0.7 Ω) during the reduction is achieved at 0.10 V. It is noted that the minimum *R_CT, Re_* of the NMP electrode (Fig. 4d) are higher than that of the FP electrode (Fig. 4c).

The smaller specific surface area of NMP compared to FP could be the main reason behind the higher minimum *R_CT, Re_*. The charge transfer resistance is related to the proton-electronic transfers within the electrode material and on the electrode-electrolyte interface, which could be affected by the crystal structure and specific surface area of the active material. As reflected by the XRD analysis, the FP material presents an amorphous crystal structure (Fig. S7), while NMP initially presents a highly crystalline characteristic (Fig. 2d). Interestingly, *ex-situ* XRD analysis of the NMP electrode after different electrochemical charging-discharging cycles (Fig. S15) indicated that it presented a reconstruction of the crystal structure where it becomes an amorphous feature. Therefore, the significant difference in the specific surface area of the two materials (FP: 103.7 m^2^ g^−1^, NMP: 1.7 m^2^ g^−1^) could be the predominant influential factor on the *R_CT, Re_*.

### Optimization of charge transfer: an example of the NMP cathode

To improve the charge transfer of the NMP electrode, conductive carbon (acetylene black, denoted below as AB) was introduced in the synthesis process, which produced a composite material composed of NMP and conductive carbon (NMP-AB). To validate the potential of NMP by minimizing the influence of the low charge transfer of the material. A relatively high proportion of conductive carbon is applied (NMP:AB ≈ 1:2 wt/wt). Note that the insufficient electronic conductivities of the transition metal phosphates are one of the limitations of the material category. This is a challenge not only for proton batteries but also for other batteries, for example, LiFePO_4_ and LiMn_x_Fe_1−x_PO_4_ for Li-ion batteries [37,38]. Developing an advanced technique for improving the charge transfer (e.g. low-proportion surface coating of carbon [38]) would be a more practical methodology. However, this is far beyond the scope of this work.

**Figure 5.**
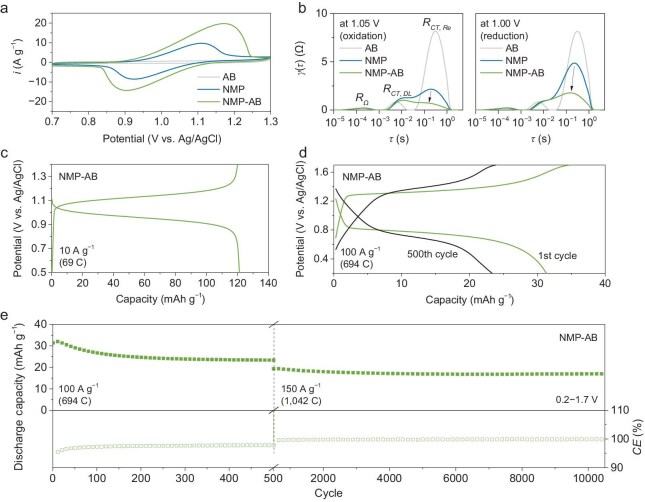
(a) CV scan with a 3-electrode configuration with NMP or NMP-AB as a working electrode at a rate of 10 mV s^−1^. (b) DRT analysis of potential at 1.05 V (vs Ag/AgCl) during charging and at 1.00 V during discharging for the NMP and NMP-AB working electrodes. (c) Galvanostatic charging-discharging potential (vs Ag/AgCl) profile of the NMP-AB electrode at 10 A g^−1^. (d) Potential profile at 100 A g^−1^ and (e) galvanostatic charging-discharging cycles of NMP-AB working electrode at 100 A g^−1^ (first 500 cycles) then at 150 A g^−1^ (subsequent 10 000 cycles).

CV scans (Fig. 5a) of the NMP-AB electrode demonstrate much higher response current density compared to that of the NMP electrode. This indicates a higher utility rate for the active component (i.e. NMP). The very low response current density of the conductive carbon (AB) indicates the negligible contribution of capacitance from AB compared to that from the active material. DRT analysis (Fig. 5b) indicates the decrease in the *R_CT, Re_* of the Mn^2+^/Mn^3+^ redox reactions for both oxidation direction (*R_CT, Re_* decreased by 66% from 2.2 to 0.7 Ω, at 1.05 V vs Ag/AgCl) and reduction direction (*R_CT, Re_* decreased by 63% from 4.9 to 1.8 Ω, at 1.00 V). Facilitated by the improved charge transfer, the NMP component in the NMP-AB composite demonstrated a discharge capacity of 121 mAh g^−1^ at 10 A g^−1^ (Fig. 5c), which is closer (84%) to the theoretical capacity of 144 mAh g^−1^ for NMP. During discharging, the electrode presented a flat voltage profile with an insignificant voltage drop (∼0.1 V, from 1.0 to 0.9 V) along with the depth of discharge before the termination of the discharging process. The NMP electrode maintained a relatively high voltage output at ∼0.8 V vs Ag/AgCl and presented a decent capacity of 31 mAh g^−1^ at 100 A g^−1^ (Fig. 5d). Even at 150 A g^−1^ (equivalent to 1042 C, 1 C = 144 mA g^−1^ for NMP), the NMP electrode still presented a capacity of ∼20 mAh g^−1^ and good cycling stability with a retention rate of 89% over 10 000 cycles (Fig. 5e and Fig. S16), and a capacity degradation rate of 0.001% per cycle. It is noted that the Coulombic efficiency (CE) was lower than 100%. The capacity degradation could be attributed to the partially irreversible Mn redox on the NMP surface, as revealed by the *ex-situ* XPS analysis (Fig. S10).

### Validation of design in quasi-solid-state proton batteries

**Figure 6.**
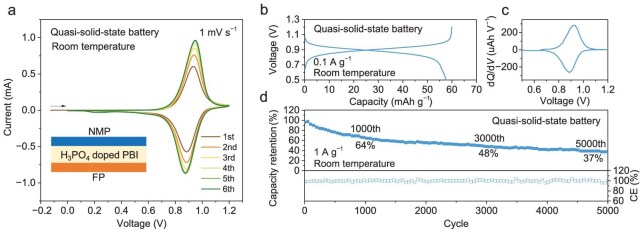
Performance of the quasi-solid-state all-phosphate proton battery. (a) CV scans of the full battery, inset image presents the FP | H_3_PO_4_ doped PBI | NMP battery configuration. (b) Galvanostatic charging-discharging voltage profile at 0.1 A g^−1^. (c) Corresponding d*Q*/d*V* profile and (d) galvanostatic charging-discharging cycles at 1 A g^−1^.

In this work, a quasi-solid-state electrolyte, i.e. H_3_PO_4_-doped PBI membrane, was utilized to further alleviate the loss of transition metal and to reduce side reactions relevant to the water component (e.g. OER) in the liquid electrolyte. To demonstrate the all-phosphate proton battery configuration, a full battery (inset image of Fig. 6a) with the FP anode and the NMP (pristine sample) cathode was evaluated at room temperature. Polybenzimidazole (PBI) film has the capability of confining the H_3_PO_4_ and has good proton conductivity (1.9 × 10^−2^ S cm^−1^ at room temperature) [39]. The H_3_PO_4_-doped PBI was previously applied in proton exchange membrane fuel cells (PEM-FCs). As a full battery with an initially H^+^-rich state of the cathode (NMP) and H^+^-deficient state of the anode (FP), the battery requires charging and is then followed by discharging (chemistry demonstrated in Fig. 1b). CV scans of the battery (Fig. 6a) at 1 mV s^−1^ demonstrate a couple of oxidation and reduction peaks centred at ∼0.9 V. These voltages of the full battery align with the CV scan result from the 3-electrode cells, where NMP and FP have redox peaks centred at ∼1 V and 0.1 V, respectively (Fig. 1c). The battery reached a discharge capacity (based on the weight of NMP) of nearly 58 mAh g^−1^ at 0.1 A g^−1^ (Fig. 6b). The corresponding d*Q*/d*V* profile (Fig. 6c, converted from the voltage-capacity profile) presents peaks that align with the CV scans. The quasi-solid-state battery also demonstrates good discharge capacity at varied current densities from 0.1 A g^−1^ (58 mAh g^−1^) to 5 A g^−1^ (36 mAh g^−1^, Fig. S17), and decent discharging capacity retention rates of 64% for 1000 cycles (Fig. 6d). To identify the possible reasons behind the capacity degradation, EIS of the quasi-solid-state full battery before (electrochemically activated for 4 CV cycles) and after 500 charging-discharging cycles were analyzed. As indicated in Fig. S18a, the total resistance of the battery after 500 cycles was slightly worse than that before electrochemical cycling. Detailed analysis using the DRT (Fig. S18b) indicates that the resistance at the high-frequency range (omics resistance and electrolyte-electrode interfaces, R_Ω_+R_I_) has increased. Note that in a solid-state battery, the resistance contributions from the solid-state electrolyte (*R_Ω_*) and the interface (*R_I_*) are difficult to separate. The increased R_Ω_+R_I_ indicates the deterioration of the electrolyte membrane or the electrolyte-electrode interface. Besides the change of R_Ω_+R_I_, a decreased *R_CT, DL_* and an increased *R_CT, Re_* was observed. The decreased *R_CT, DL_* could be related to the reconstruction/redistribution of the conductive framework, while the increased *R_CT, Re_* could be related to the deterioration of the electrode material after the charging-discharging cycle. After 5000 cycles, the capacity retention rate remains at 37% with an average capacity degradation rate of 0.0126% (per cycle) and an average CE of 99.8% (Fig. 6d). The self-discharging evaluations (Fig. S19) of the battery indicate that the discharge capacity retention rates of the charged battery after 12 hour and 24 hour rests were 50% and 27%, respectively, corresponding to a self-discharging rate of 50% and 73% (∼3% per hour), respectively. The performance of the quasi-solid-state battery in this work is comparable to recently reported advanced solid-state proton batteries and presents superior high-rate performance at room temperature (Table S1) [17,18,40–42]. The highly reversible characteristics, good electrochemical performance and decent cycle stability validate the concept of the all-phosphate proton battery demonstrated in Fig. 1b.

## CONCLUSION

An all-phosphate configuration with both phosphate electrodes and a phosphoric acid electrolyte has been proposed to open up an avenue for developing a promising category of inorganic transition metal phosphate electrodes for rechargeable proton batteries. The battery with a FP anode (as an example of Fe^2+/3+^ redox couple) and a NMP cathode (as an example of Mn^2+/3+^ redox couple) demonstrates good reversibility and provides a potential gap of ∼0.9 V, which shows promise in forming a full proton battery. *In-situ* XAS characterizations capture the electronic and structural variations during the H^+^ insertion and de-insertion process. *In-situ* DRT analysis reveals the resistance of the FP and NMP is attributed mainly to charge transfers, which are dependent on the potential during the electrochemical oxidation/reduction process. Optimization of the electronic transfers via forming a composite (NMP-AB) addresses the limitation of low electronic conductivity of the phosphate material, leading to reduced charge transfer resistances and improved active species utilization. The NMP-AB electrode presented a flat voltage profile with an insignificant voltage drop (∼0.1 V) along with the depth of discharge. The NMP-AB electrode also demonstrates a good discharge capacity of 121 mAh g^−1^ at 10 A g^−1^ and good cycle stability for 10 000 cycles at high current densities up to 150 A g^−1^ (equivalent to 1042 C). By applying a quasi-solid-state electrolyte, a full proton battery with the all-phosphate configuration demonstrates a decent capacity and good stability for 5000 cycles operating at room temperature. The battery shows an average capacity degradation rate of 0.0126% (per cycle) and an average CE of 99.8%. The discovery contained in this work advances the field of proton battery research by opening an avenue for developing a large category of phosphate materials. This helps to address the research gap of insufficient material systems and material options, especially as cathodes of proton batteries. Moreover, this work addresses the missing methodologies for screening of redox couples, design of transition metal compounds, synthesis and evaluation of the transition metal phosphates, and matching them in a full proton battery.

## MATERIALS AND METHODS

### Preparation of electrode materials

The phosphate materials were prepared via a precipitation method. To prepare the NH_4_MnPO_4_·H_2_O, (NMP), 5.60 g MnSO_4_^.^H_2_O was dissolved in deionised water to prepare a salt solution with a concentration of 2 M. A 70 mL base solution (pH = 7) was prepared using ammonia solution (pH buffer, 4 M NH_4_OH) and diluted phosphoric acid solution (precipitating agent, 2 M H_3_PO_4_), which contains a stoichiometric amount (16.5 mL) of PO_4_^3−^ to facilitate the participation of NMP. The prepared Mn-salt solution was carefully syringe-injected into the base solution while simultaneously injecting 4 M NH_4_OH solution to maintain the pH at around 7 for NMP precipitation. The reaction system was carefully controlled by temperature (50°C), pH (6.8 to 7.0), atmosphere (inert, protected by N_2_), stirring speed (500 rpm) and time (3 hours) to ensure the complete precipitation of Mn^2+^. The product was separated and washed with deionised water using a centrifuge. The obtained solid with a light pink colour was dried at 100°C in an oven overnight.

FePO_4_·2H_2_O (FP) was prepared with a similar wet-precipitation method modified from the literature [43]. Specifically, 8.04 g Fe(NO_3_)_3_·9H_2_O was reacted with 10 mL 2 M H_3_PO_4_ solution at a controlled temperature of 25°C, pH range of 2.4 to 2.5, and a stirring speed of 500 rpm in ambient air. The product (FP) was separated and washed with the same method as that for NMP. The resulting solid with a light orange colour was dried at 60°C in an oven overnight.

### Preparation of electrodes

Free-standing electrode sheets containing the transition metal phosphate active materials (e.g. FP or NMP), acetylene black (AB) conductive additive, and polytetrafluoroethylene (PTFE) binder were prepared with a weighting ratio of 7:2:1. For example, to prepare the NMP cathode sheet, 0.07 g NMP, 0.02 g AB, 1.67 g 0.6 wt% PTFE solution (containing 0.01 g PTFE, dissolved in ethanol and water mixture), and an additional 5 mL ethanol were mixed using an ultrasonic bath for 0.5 hours. The mixture was dried thoroughly in an oven at 60°C and the resultant powder was gathered. A few drops of absolute ethanol were used to re-wet the powder, followed by repeatedly pressing using a spatula until gum was formed. The gum was pressed into a thin sheet by a glass roller to target thickness. The sheet was dried at 60°C in an oven. The disk electrode was cut from the sheet using a hole puncher.

### Preparation of the quasi-solid-state electrolyte and assembling of quasi-solid-state full batteries

The quasi-solid-state electrolyte, i.e. phosphoric acid (H_3_PO_4_) doped poly(aryletherbenzimidazole) (O-PBI) was prepared by PBI membrane preparation, acid uptake and drying. A dry PBI membrane was prepared by solution casting as mentioned in our previous work [44,45]. The dry PBI membrane was then immersed in 85% H_3_PO_4_ at 60°C overnight. Before assembling the quasi-solid-state batteries, the H_3_PO_4_-doped PBI membrane was dried at 160°C for 30 min to remove residual water. A coin-type quasi-solid-state full battery composed of a FP anode, a H_3_PO_4_ doped PBI electrolyte film, and a NMP cathode was constructed. The FP anode (Φ 1.0 cm, area loading of FP: ∼1 mg cm^−2^) and the NMP cathode (Φ 0.8 cm, area loading of NMP: ∼1 mg cm^−2^) were pressed on two sides of a dried H_3_PO_4_-doped PBI electrolyte membrane (Φ 1.9 cm) using a hydraulic press (gauge pressure 2 MPa). The sandwich electrode-electrolyte-electrode component was then transferred into an Ar-filled glove box and sealed in a CR2032 battery case with a stainless-steel plate and a stainless-steel ring to ensure sufficient contact of all components. To evaluate the self-discharging capacity retention rate, the full batteries were first fully charged to 1.2 V, then rested for 0.5, 1, 2, 4, 12 and 24 hours [20].

Other experimental details are available in the Supplementary Data, including preparation of composite electrode materials, material characterizations, setup of 3-electrode cells, electrochemical evaluation, *in-situ* electrochemical characterization and *in-situ* quick-scan X-ray absorption measurements.

## Supplementary Material

nwaf226_Supplemental_File
