# Supplementary material for: Design methodology of a promising category of metal phosphate electrodes for quasi-solid-state proton batteries
Source: Natl Sci Rev. 2025 May 31;12(7):nwaf226. doi: 10.1093/nsr/nwaf226 (PMC12225172; doi:10.1093/nsr/nwaf226)
Supplement: nwaf226_Supplemental_File [file nwaf226_supplemental_file.pdf]

SUPPLEMENTARY DATA

Design methodology of a promising category of metal phosphate electrodes for quasi-solid-state proton batteries

Yijun Zhong[1], Leqi Zhao[1], Daqin Guan[1], Zehua Wang[1], Hongwei Wu[1], Jingyuan Liu[2] and Zongping Shao[1,*]

[1]Curtin Centre for Advanced Energy Materials and Technologies, WA School of Mines: Minerals, Energy and Chemical Engineering (WASM-MECE), Curtin University, Perth, Western Australia, 6102, Australia;

[2]Altech Batteries Ltd, Subiaco, Western Australia, 6008, Australia

∗**Corresponding author**. E-mail: zongping.shao@curtin.edu.au

Supplementary Methods

Preparation of composite electrode materials

Transition metal phosphate–carbon composite materials were prepared using a one-pot protocol. Take the NMP-C as an example, a 750 mL base solution (containing the stoichiometric amount of 2 M H_3PO_4 and 4 M NH_4OH) was first prepared and adjusted to pH=7, followed by stirring under N_2 flow protection at 50 ℃ and 700 rpm for 20 min to initiate the precipitation process. Subsequently, 200 mL conductive carbon suspension (containing 1 g conductive carbon and 0.2 g polyvinylpyrrolidone (PVP) dispersion agent) was added, quickly followed by the addition of 50 mL of $MnSO_4$ solution (containing 0.56 g $MnSO_4 \cdot H_2O$). The mixture was stirred thoroughly, followed by separation and washing steps similar to those for preparing pure NMP. The obtained product was denoted NMP-AB.

For free-standing electrode sheet with transition metal phosphate-carbon composite (e.g., NMP-AB), no additional conductive carbon was introduced during the electrode sheet preparation. The NMP-AB and PTFE binder has a weight ratio of 8:2.

Material characterizations

The crystal structure of the samples was analysed with XRD (Bruker D8 Advance equipped with a Cu X-ray source). The morphology of the samples was observed using SEM (ZEISS NEON 40EsB). EDS elemental mapping and SAED of the sample were obtained using a TEM (FEI Talos FS200X G2). The chemical states of the samples were evaluated using XPS (Kratos AXIS Ultra DLD, Al Kα radiation). The specific surface areas (estimated via Brunauer–Emmett–Teller (BET) theory) and total pore volume (at p/p_0=0.95) of the samples were evaluated by N_2 adsorption-desorption at 77 K using Micromeritics TriStar. Pore size distributions were calculated using the adoption branch of the isotherm data and by the Barrett–Joyner–Halenda (BJH) method. TGA data were recorded and analysed using a simultaneous thermal analyser (TA Instruments SDT Q600). The instrument was calibrated and operated under a N_2 atmosphere, with measurements conducted up to 700 ℃ at a heating rate of 10 ℃ min^{-1}, with 20 mg sample placed in an alumina crucible.

Setup of 3-electrode cells

A static 3-electrode cell is composed of a working electrode (e.g., FP electrode), a reference electrode (Ag/AgCl) and a counter electrode (graphite stick). 20 mL 85 wt.% phosphoric acid (in H_2O) was used as an electrolyte. The as-prepared electrode disk (Φ 0.8 cm, areal active material loading is ~1 mg cm^{-2}) was rolled on the surface of a graphite sheet current collector (1×2 cm).

Electrochemical evaluation

The 3-electrode cell and 2-electrode coin-type full batteries were evaluated at room temperature (~23 ℃). The electrode performance evaluation and electrochemical characterisations with the 3-electrode cell were performed using a Biologic VSP potentiostat. The current density and capacity were calculated based on the mass loading of the active materials (e.g., NMP) on the working electrode, excluding the current collector (e.g. graphite paper), the conductive carbon and the binder. The performance of the quasi-solid-state full batteries was evaluated using a LANHE battery tester. The current density and capacity were calculated based on the mass loading of the active materials on the cathode (i.e., NMP).

***In-situ* electrochemical characterisation**

In-situ DRT analysis using the data from SPEIS was applied to characterise the resistance of the working electrode at different potentials in a 3-electrode setup. The impedance data were obtained using a Biologic VSP potentiostat. Take the FP electrode for example, an interval potential of 50 mV was first applied from 0.3 to -0.2 V vs. Ag/AgCl (discharging) and then reversed from -0.2 to 0.3 V (charging). A constant potential was kept for 1 min at each potential level, followed by obtaining the EIS data (potential variation at 10 mV, frequency from 10^5 to 1 Hz). For NMP, similar parameters were utilised, except for the range of potential (NMP: 0.7 to 1.3 V) and the sequence of the staircase (NMP: first charging then discharging). DRT analysis of the impedance data was conducted using DRTtools.[1] The quality of impedance data was validated with the Kramers-Kronig test by Lin-KK Tools,[2–4] where the error over the frequency range was less than 1%. Examples of Lin-KK error analysis for the data present in **Figure 4c&d** and **Figure 5b** are shown in **Figure S20-22**.

***In-situ* quick-scan X-ray absorption measurements**

The Fe and Mn K-edge XAS were recorded in a transmission mode at TPS BL-44A beamline station in NSRRC, Taiwan, China. The collection time for each spectrum was 5 minutes. The spectra were

recorded in a three-electrode setup with a self-assembly cell. Kapton tape was utilised to seal the *in-situ* cell window, which allowed X-rays to be transmitted through the window and the electrode, and to reach the detector for *in-situ* XAS spectra recording. 15 mg active material (i.e., FP, or NMP) powders were mixed with 3 mg conductive carbon and were dispersed in 200 μL ethanol containing 50 μL Nafion solution (5%). The mixture was sonicated for 20 min. The mixture ink was drop-cast onto a 1x1 cm^2 carbon cloth to produce a working electrode. A glassy carbon electrode was used as the counter electrode. A Ag/AgCl electrode was used as the reference electrode. 85% H_3PO_4 was used as the electrolyte.

Supplementary Figures

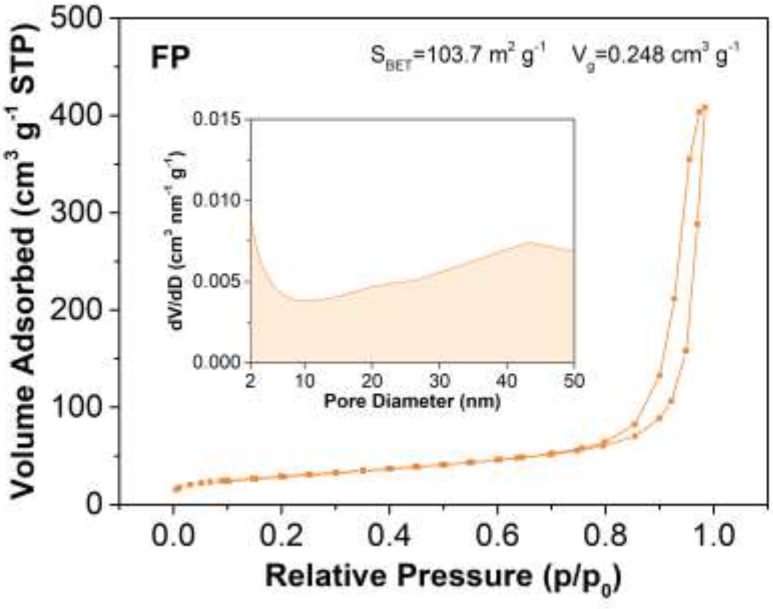

Figure S1. N_2 adsorption-desorption isotherm at 77 K of FP, the inset image presents the BJH pore size distribution from the adsorption branch.

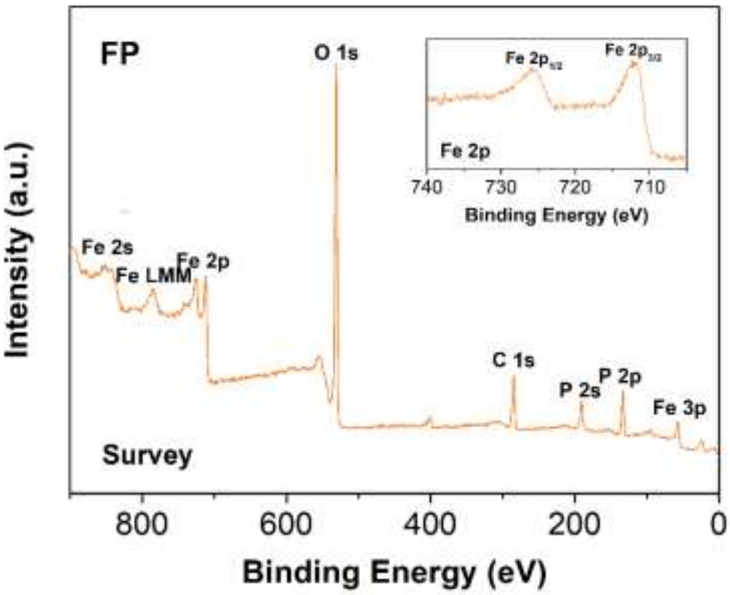

Figure S2. XPS survey spectrum of FP, the inset image presents the Fe 2p spectrum.

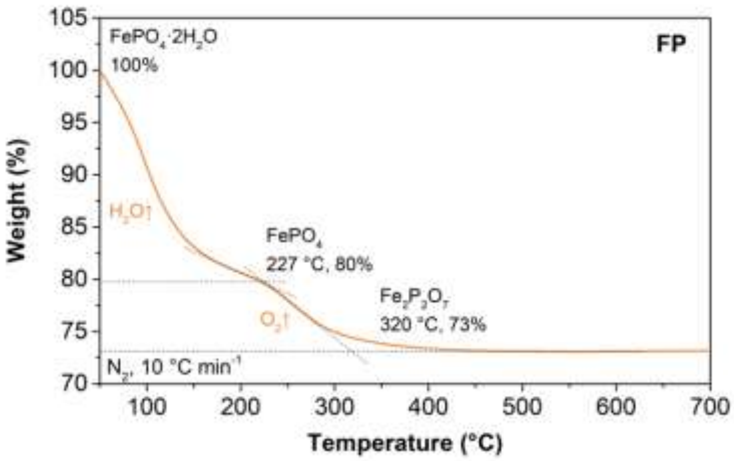

Substance	Molar Mass g mol^{-1}	Mass Ratio vs FP
$FePO_4 \cdot 2H_2O$ (FP)	186.85	100%
$FePO_4$	150.82	80%
$Fe_2P_2O_7$	285.63	76%

Figure S3. TGA profile of FP under N_2 flow.

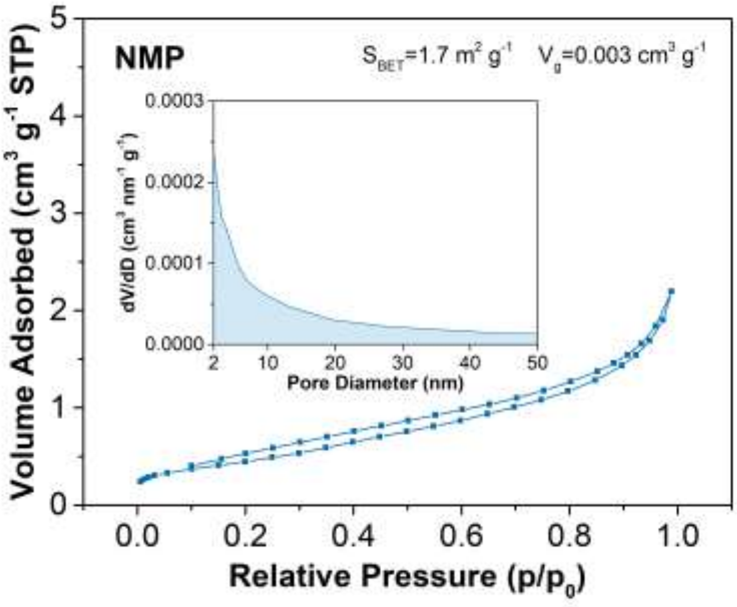

Figure S4. N_2 adsorption-desorption isotherm at 77 K of NMP, the inset image presents the BJH pore size distribution from the adsorption branch.

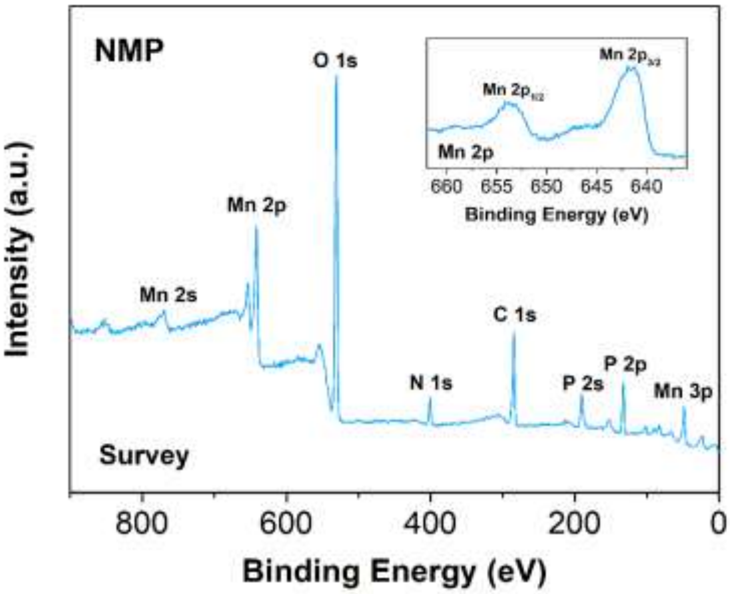

Figure S5. XPS survey spectrum of NMP, the inset image presents the Mn 2p spectrum.

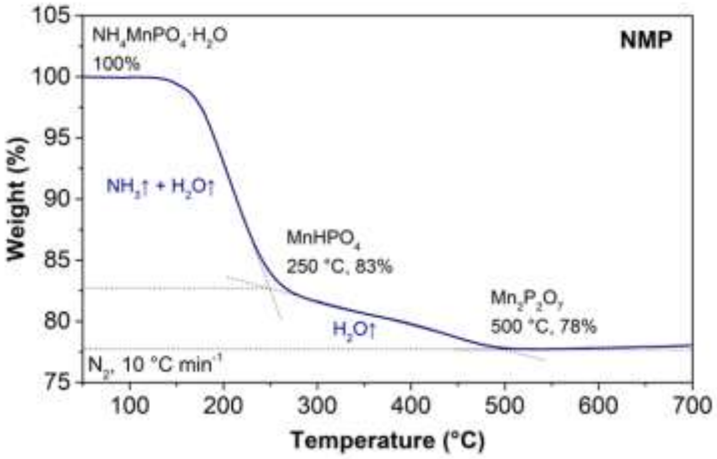

Substance	Molar Mass g mol^{-1}	Mass Ratio vs NMP
$NH_4MnPO_4 \cdot H_2O$ (NMP)	185.96	100%
$MnHPO_4$	150.92	81%
$Mn_2P_2O_7$	283.82	76%

Figure S6. TGA profile of NMP under N_2 flow.

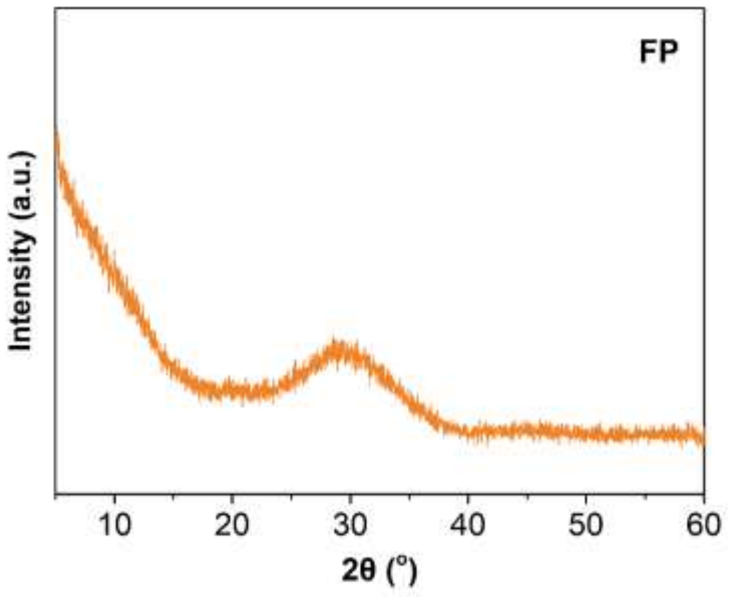

Figure S7. XRD profile of FP.

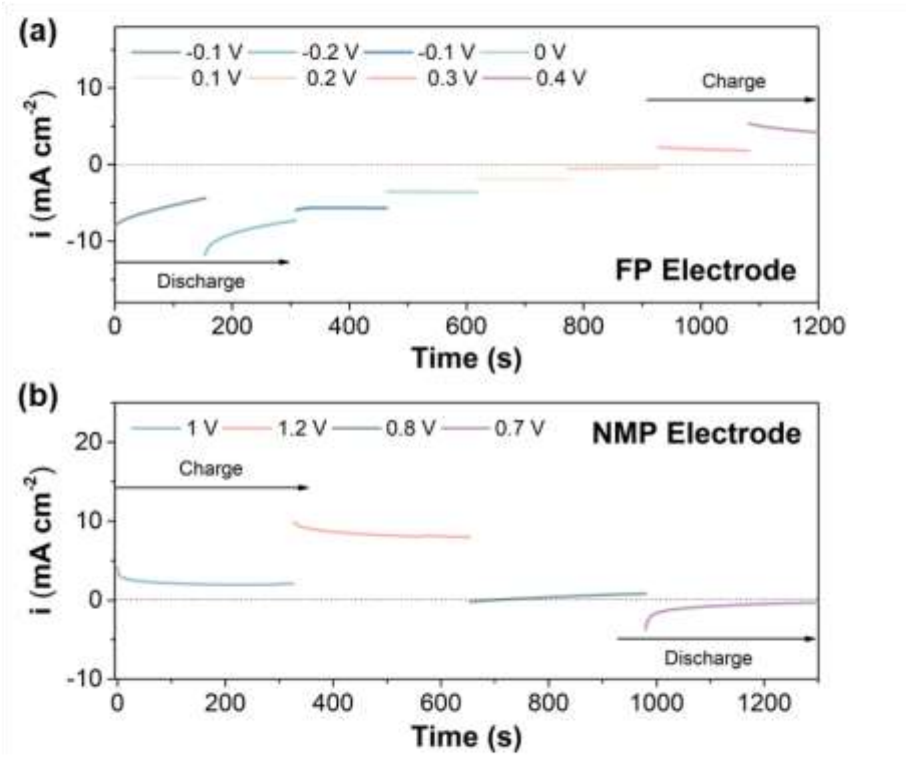

Figure S8. *i–t* curves of 3-electrode configuration cell for the *in-situ* XANES measurements: (a) with a FP working electrode and (b) with a NMP working electrode.

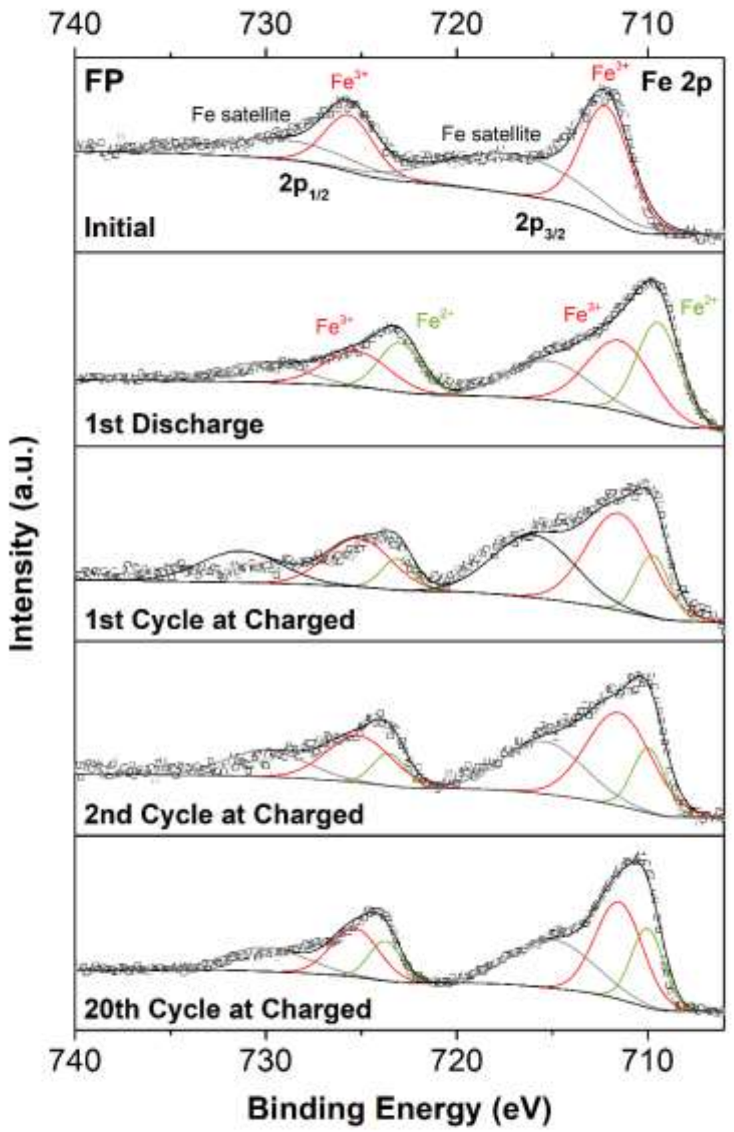

Figure S9. XPS Fe 2p spectra of FP at the initial state, after 1st discharge, after 1st cycle (charged state), 2nd cycle (charged state) and 20th cycle (charged state).

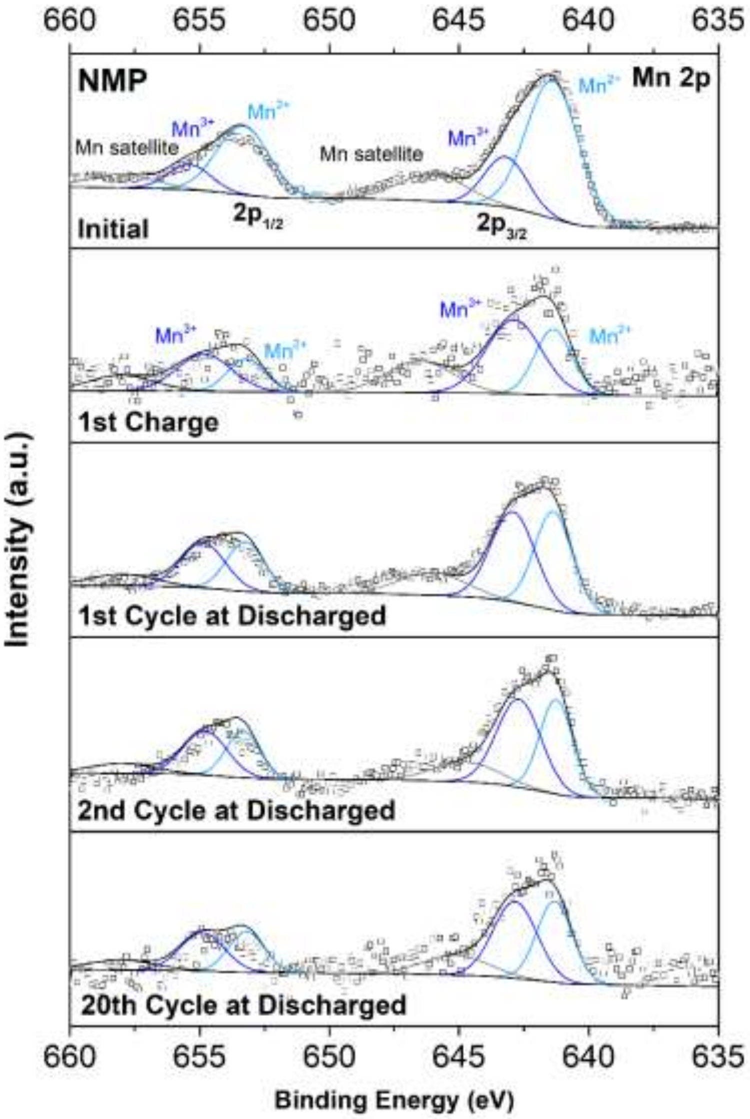

Figure S10. XPS Mn 2p spectra of NMP at the initial state, after 1st charge, after 1st cycle (discharged state), 2nd cycle (discharged state) and 20th cycle (discharged state).

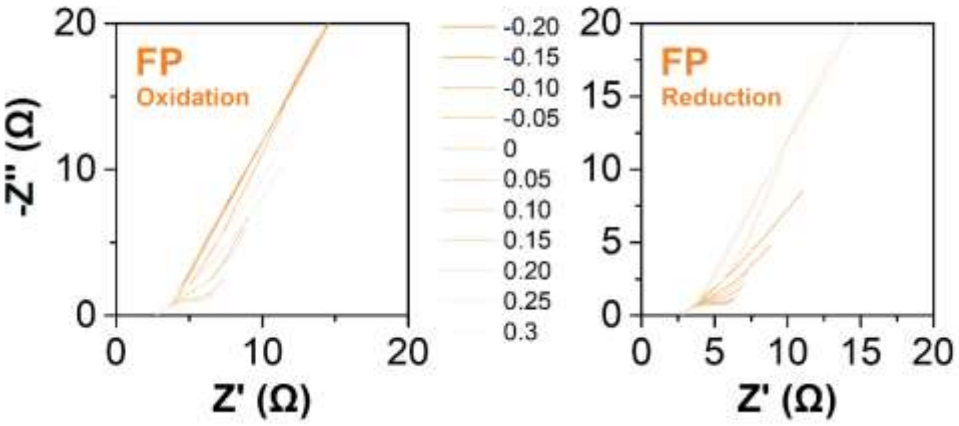

Figure S11. Nyquist plots of FP at different potentials for the DRT analysis.

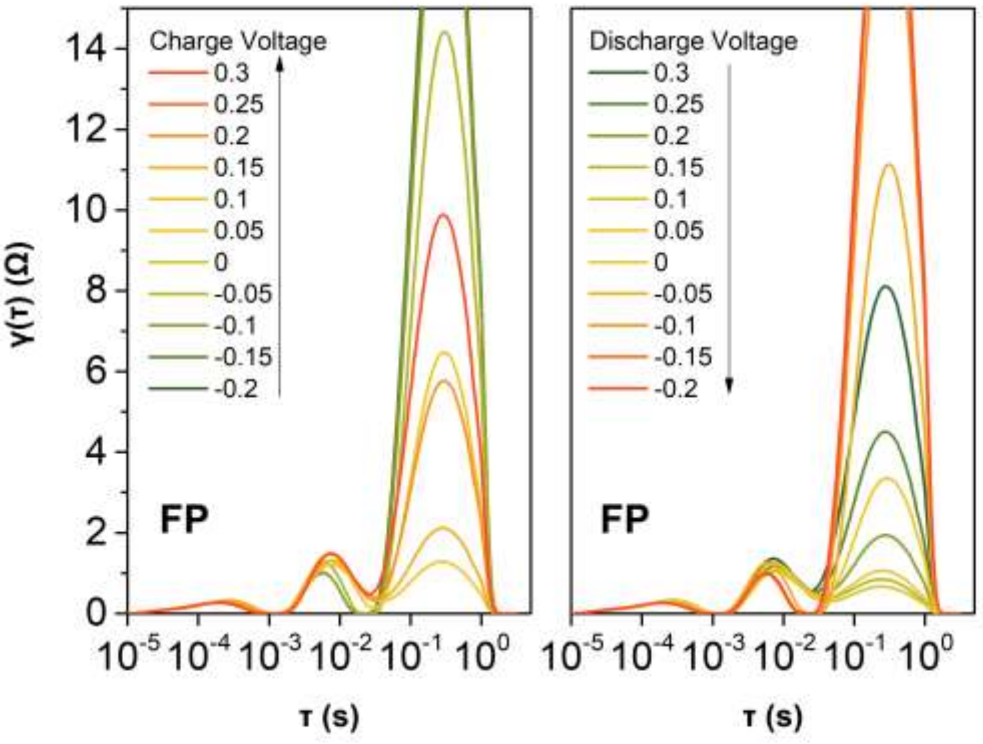

Figure S12. DRT analysis from the data of **Figure S11** for FP.

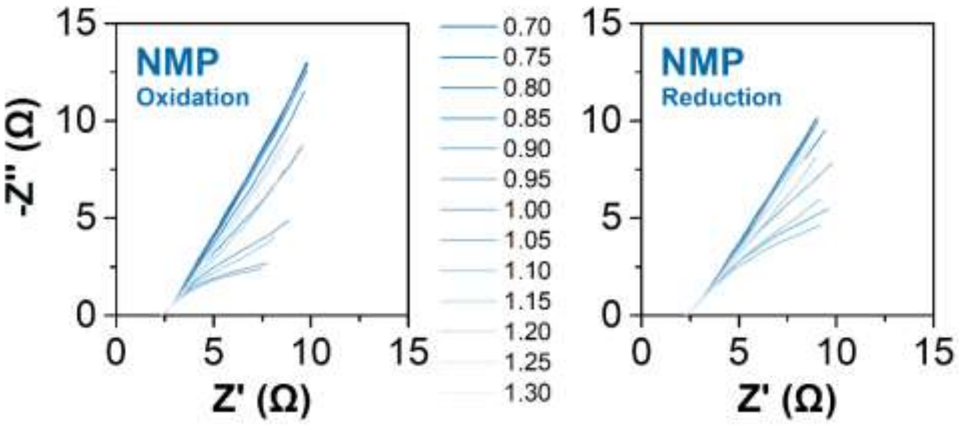

Figure S13. Nyquist plots of NMP at different potentials for the DRT analysis.

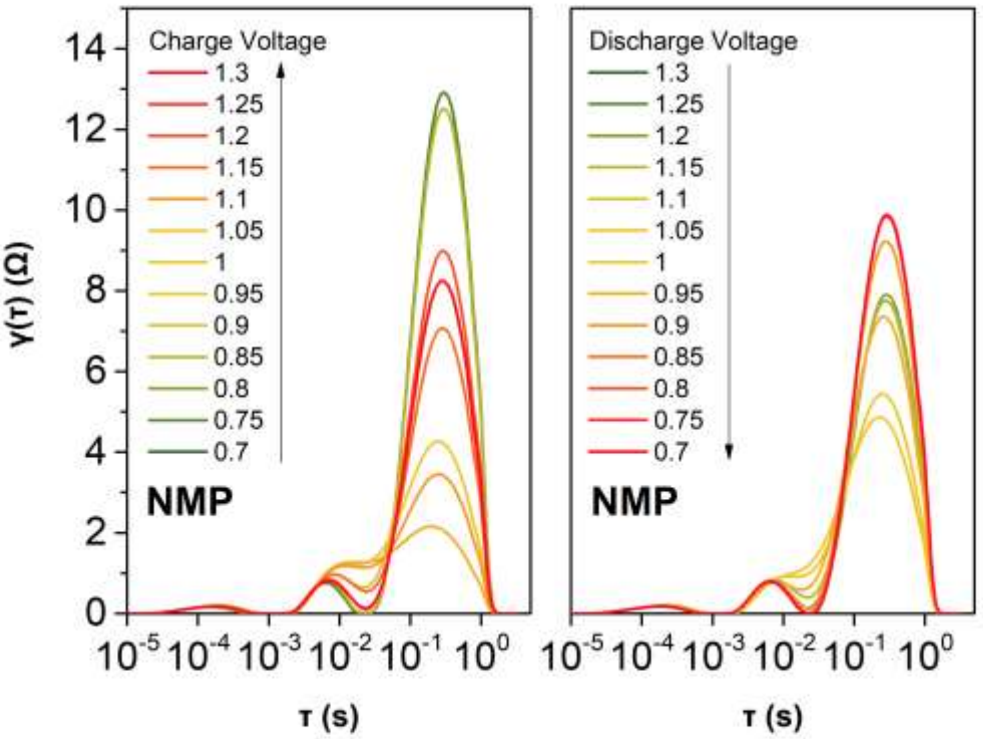

Figure S14. DRT analysis from the data of **Figure S13** for NMP.

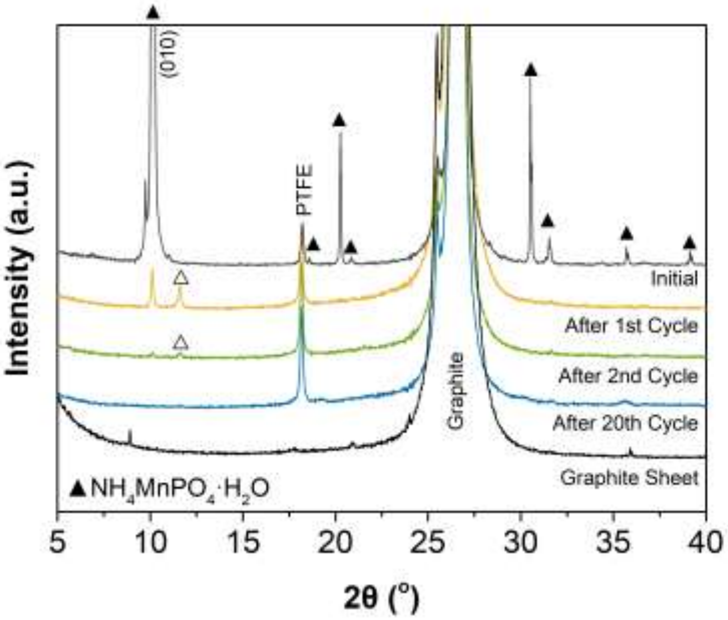

Figure S15. XRD profiles of NMP electrode at the initial state, after 1st cycle, after 2nd cycle and after 20th cycle.

The XRD of the NMP electrode after 1st cycle indicates a new peak centred at 2θ≈11.6° is identified near the peak representing the (0 1 0) facet of NMP centred at the lower angle of 2θ≈10.1°. The new peak at a higher angle (smaller d-spacing) could be associated with the insertion of H^+ (or in the form of H_3O^+ with ionic radius: 1.0 Å)[5], which has a smaller radius than that of NH_4^+(1.5 Å)[6], during the first cycle. The further comparison of the XRD profile of the initial sample and 1st, 2nd and 20th cycles indicated that the NMP electrode presented reconstruction of the crystal structure where the well-defined crystalline feature became an amorphous feature after electrochemical charging-discharging cycles.

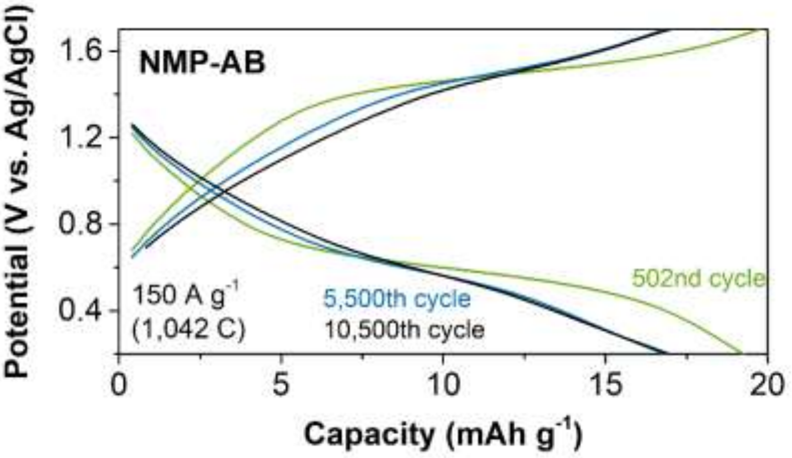

Figure S16. Galvanostatic charging-discharging potential (vs. Ag/AgCl) profile of NMP-AB working electrode at 150 A g^{-1}, data extracted from **Figure 5e**.

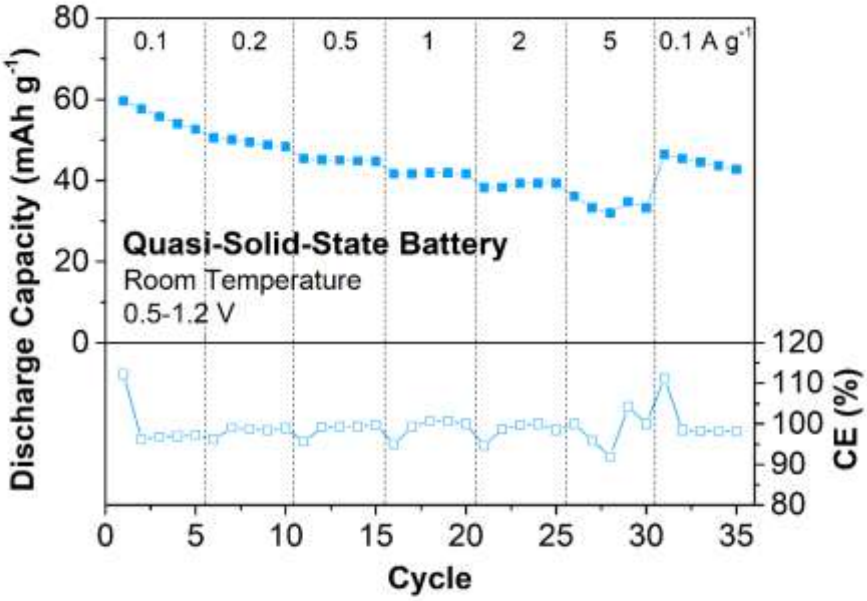

Figure S17. Galvanostatic discharging capacities and Coulombic efficiencies at different current densities of the quasi-solid-state battery (FP | H_3PO_4 doped PBI | NMP) tested at room temperature.

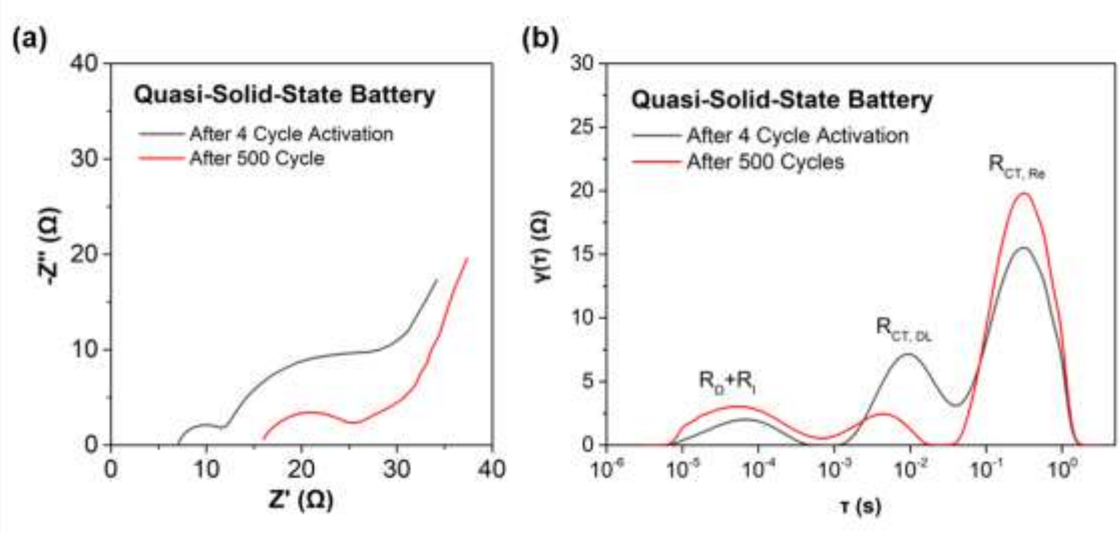

Figure S18. (a) Nyquist plots and (b) corresponding DRT analysis of a quasi-solid-state full battery after activation and after 500 charging-discharging cycles.

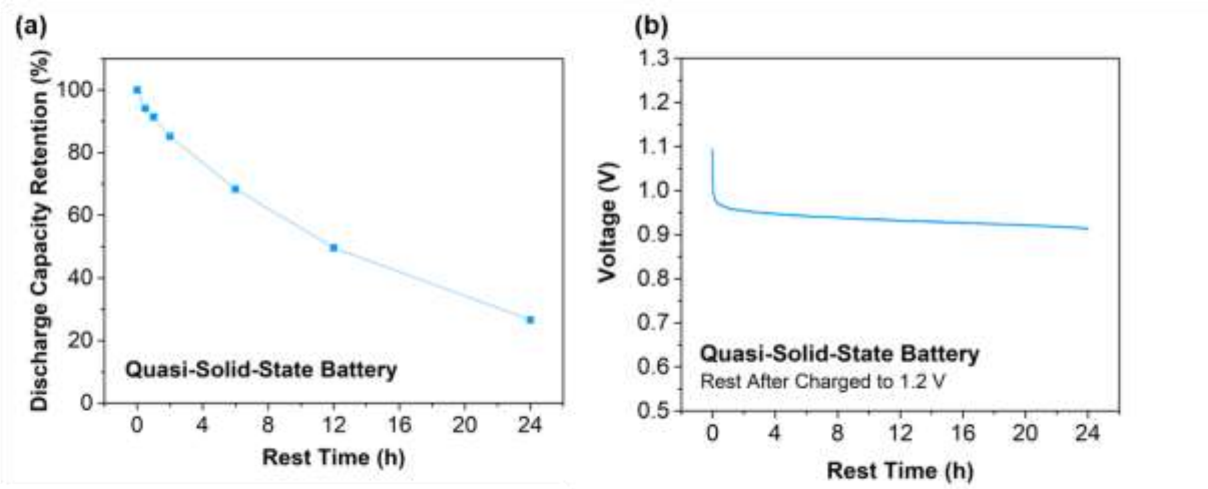

Figure S19. (a) Self-discharging capacities at different rest durations and (b) the variation of open circuit voltage after charging to 1.2 V of the quasi-solid-state proton battery with NMP cathode, FP anode and the quasi-solid-state electrolyte.

As another indication of self-discharging, the open circuit voltage (OCV) of a fully charged battery was also monitored for 24 h (**Figure S19b**). The OCV presented a quick drop from 1.20 V to 1.00 V in the first 2 min, which could be associated mainly with the ohmic drop after the de-polarisation. The OCV further drops gradually in the next 24 h from 1.00 V to 0.91 V.

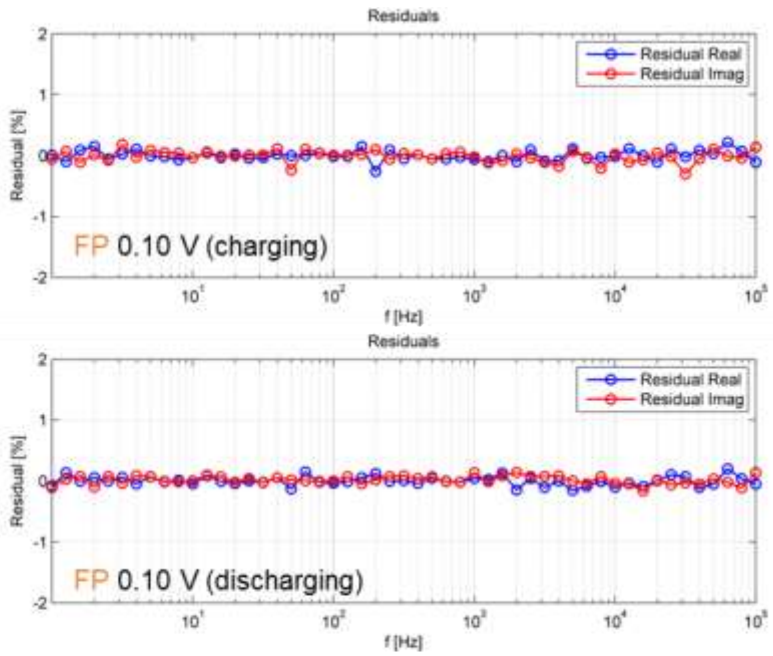

Figure S20. Lin-KK validity test result of the SPEIS impedance data (charging at 0.10 V and discharging at 0.10 V, vs. Ag/AgCl) of FP.

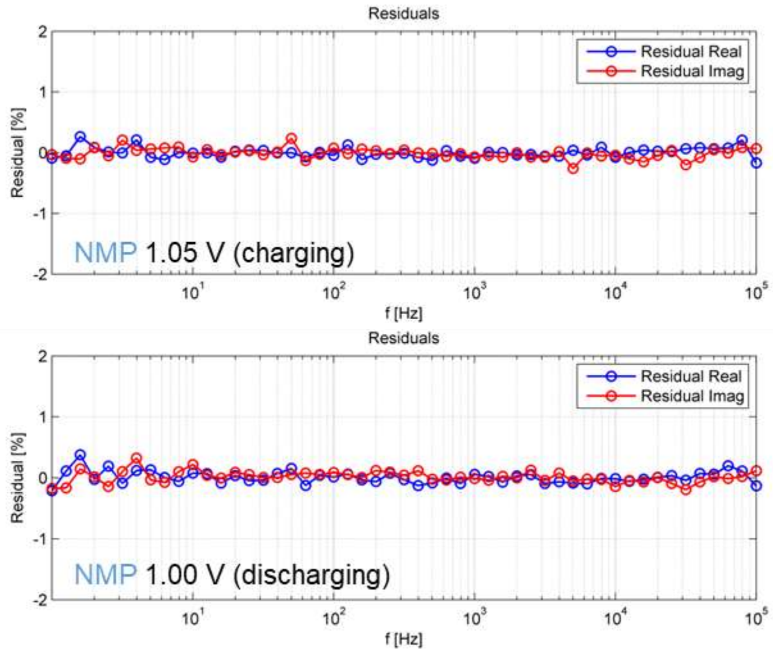

Figure S21. Lin-KK validity test result of the SPEIS impedance data (charging at 1.05 V and discharging at 1.00 V, vs. Ag/AgCl) of NMP.

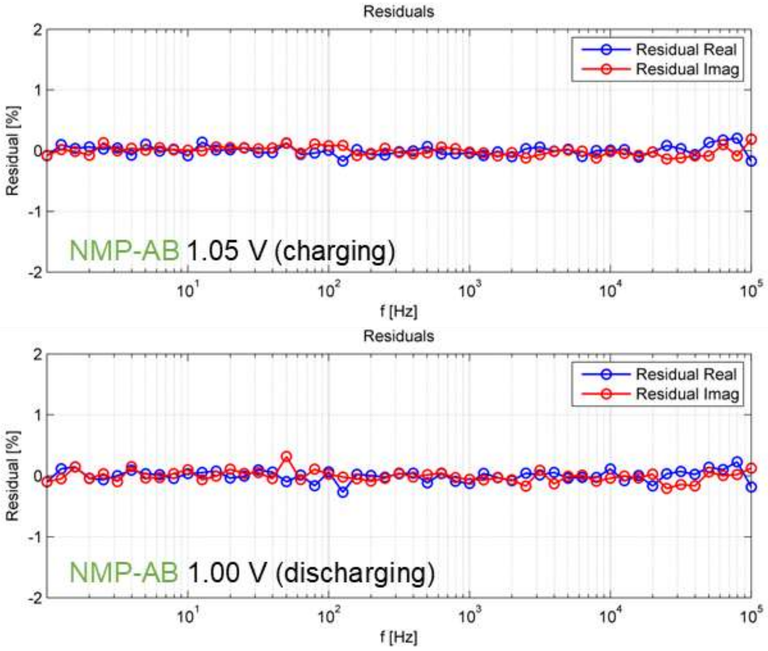

Figure S22. Lin-KK validity test result of the SPEIS impedance data (charging at 1.05 V and discharging at 1.00 V, vs. Ag/AgCl) of NMP-AB.

Supplementary Table

Table S1. Comparison of composition and performance parameters of solid-state proton batteries.

Cathode //Anode	Electrolyte	Voltage window (V)	Capacity (mAh g^{-1} @A g^{-1})	Rate (mAh g^{-1} @A g^{-1})	Cycle life Retention% @cycles	[Ref.] Year
$NH_4MnPO_4 \cdot H_2O$ //$FePO_4 \cdot 2H_2O$	H_3PO_4-PBI	0.5–1.2	58 @0.1 based on cathode	36 @5	64% @1000 37% @5000	This work
$K_2NiFe(CN)_6$ //MoO_3	MeSA@PBI-COF	0.1–1.6	57 @0.5 based on cathode	42 @2	74% @8000	[7] 2024
HDC //activated carbon	PVA-H_2SO_4	-0.3–0.75	50 @0.1 based on cathode	32 @1	90% @2000	[8] 2022
$LiVPO_4F$ //MoO_3	Polyphosphoric acid	0–1.3	40 @0.05 based on cathode and anode	27 @9.3 (100 ℃)	~100% @1000	[9] 2022
$Cu^{II}[Fe^{III}(CN)_6]_{2/3} \cdot 4H_2O$ //MoO_3	$[Zn_3(H_2PO_4)_6(H_2O)_3]$ (1,2,3-benzotriazole)	0–1.2	55 @0.01 based on cathode	12 @0.05	~75% @1000 (100 ℃)	[10] 2021
MnO_2@graphite felt //MoO_3	frozen (-70 ℃) 2M H_2SO_4+2M $MnSO_4$	0.8–1.55	172 @0.1 based on anode (-70 ℃)	~120 @0.5 (-70 ℃)	~100% @100 (-70 ℃)	[11] 2020

Note:

Performance data is reported at room temperature unless specified.

MeSA: methanesulfonic acid; PBI: polybenzimidazole; COF: covalent-organic framework

HDC: 2,5-dichloro-1,4-phenylene bis((ethylsulfonyl)amide); PVA: polyvinyl alcohol

References for Supplementary Data

1. Wan TH, Saccoccio M, Chen C *et al.* Influence of the discretization methods on the distribution of relaxation times deconvolution: implementing radial basis functions with DRTtools. *Electrochim Acta* 2015; **184**: 483–99.
2. Boukamp BA. A linear Kronig‐Kramers transform test for immittance data validation. *J Electrochem Soc* 1995; **142**: 1885.
3. Schönleber M, Klotz D, Ivers-Tiffée E. A method for improving the robustness of linear Kramers-Kronig validity tests. *Electrochim Acta* 2014; **131**: 20–7.
4. Schönleber M, Ivers-Tiffée E. Approximability of impedance spectra by RC elements and implications for impedance analysis. *Electrochem Commun* 2015; **58**: 15–9.
5. Wang X, Bommier C, Jian Z *et al.* Hydronium-ion batteries with perylenetetracarboxylic dianhydride crystals as an electrode. *Angew Chem Int Ed* 2017; **56**: 2909–13.
6. Han J, Varzi A, Passerini S. The emergence of aqueous ammonium-ion batteries. *Angew Chem Int Ed* 2022; **61**: e202115046.
7. Ren X-Y, Song J-B, Zhang G-Q *et al.* Covalent-organic framework with superior proton conduction for solid-state proton battery application. *ACS Mater Lett* 2024; **6**: 4036–41.
8. Shen D, Rao AM, Zhou J *et al.* High-potential cathodes with nitrogen active centres for quasi-solid proton-ion batteries. *Angew Chem Int Ed* 2022; **61**: e202201972.
9. Liao M, Ji X, Cao Y *et al.* Solvent-free protic liquid enabling batteries operation at an ultra-wide temperature range. *Nat Commun* 2022; **13**: 6064.
10. Ma N, Kosasang S, Yoshida A *et al.* Proton-conductive coordination polymer glass for solid-state anhydrous proton batteries. *Chem Sci* 2021; **12**: 5818–24.
11. Yan L, Huang J, Guo Z *et al.* Solid-state proton battery operated at ultralow temperature. *ACS Energy Lett* 2020; **5**: 685–91.
